# Supplementary material for: Pparg promotes differentiation and regulates mitochondrial gene expression in bladder epithelial cells
Source: Nat Commun. 2019 Oct 9;10:4589. doi: 10.1038/s41467-019-12332-0 (PMC6785552; doi:10.1038/s41467-019-12332-0)
Supplement: Supplementary file 1 — Supplementary Information [file 41467_2019_12332_MOESM1_ESM.pdf]

***Pparg* promotes differentiation and regulates mitochondrial gene expression in bladder epithelial cells**

Liu et al.

Supplementary Table 1. Gene expression changes in the urothelium of *ShhCre;Pparg$^{fl/fl}$* mutants

Squamous markers	FoldChange	pvalue	padj
Krt1	3.92	5.79E-15	4.80E-14
Krt4	30.94	2.85E-21	3.48E-20
Krt5	0.76	8.52E-02	1.32E-01
Krt6a	438.64	4.71E-16	4.17E-15
Krt6b	9.75	9.51E-02	1.45E-01
Krt13	14.70	1.31E-15	1.13E-14
Krt14	22.56	5.38E-22	6.81E-21
Krt15	5.55	4.20E-13	3.02E-12
Krt16	31.45	1.72E-03	3.98E-03

Superficial cell markers	FoldChange	pvalue	padj
Upk1a	0.40	4.29E-59	2.13E-57
Upk1b	0.30	1.09E-19	1.23E-18
Upk2	0.47	4.24E-12	2.79E-11
Upk3a	0.17	6.41E-49	2.34E-47
Upk3b	0.09	4.70E-12	3.08E-11
Krt18	0.58	2.38E-13	1.76E-12
Krt20	0.00	6.67E-27	1.05E-25
Rab27b	0.38	1.69E-32	3.29E-31
Snx31	0.50	1.39E-16	1.28E-15
Uchl1	0.05	2.89E-93	3.16E-91
Grhl3	0.58	2.47E-07	1.00E-06
Gata3	1.17	4.05E-02	6.91E-02
Foxa1	1.07	1.83E-01	2.56E-01

Tricarboxylic acid cycle	FoldChange	pvalue	padj
Aco1	0.30	3.44E-183	1.64E-180
Idh2	3.46	1.01E-34	2.16E-33
Sdhc	0.60	4.01E-11	2.39E-10
Suclg2	0.69	6.08E-09	2.92E-08

Fatty Acyl-CoA transport	FoldChange	pvalue	padj
Cpt1a	0.90	3.93E-02	6.73E-02
Cpt2	0.49	7.47E-23	9.87E-22
Slc25a20	0.56	1.91E-10	1.07E-09

ANTs (energy transfer)	FoldChange	pvalue	padj
slc25a4	0.75	6.06E-05	1.80E-04
slc25a5	0.78	1.66E-07	6.87E-07
slc25a6	0.56	6.83E-14	5.22E-13
Pdk4	0.12	3.34E-29	5.70E-28

Mito antioxidant systems	FoldChange	pvalue	padj
Sod1	0.43	8.42E-46	2.77E-44
Sod2	0.69	2.80E-11	1.70E-10
Sod3	0.06	1.34E-17	1.31E-16
Prdx3	0.43	8.91E-43	2.57E-41
Txn2	0.44	3.36E-58	1.62E-56
Txnrd2	0.68	2.23E-06	8.08E-06
Nfe2l2	0.35	2.85E-46	9.44E-45
Cat	0.40	4.05E-43	1.19E-41
Ucp1	1.36	4.00E-01	4.88E-01
Ucp2	2.02	7.46E-55	3.20E-53
Ucp3	0.09	2.27E-05	7.16E-05

* mtDNA genes	FoldChange	pvalue	padj
mt-Co1	0.70	1.28E-05	6.26E-05
mt-Cytb	0.76	3.24E-03	9.80E-03
mt-Nd4	0.77	4.86E-03	1.41E-02
mt-Nd5	0.60	4.55E-08	3.10E-07
mt-Nd6	0.60	1.07E-03	3.60E-03
mt-Rnr1	0.73	6.58E-03	1.85E-02

Oxidative phosphorylation	FoldChange	pvalue	padj
Ndufa1	0.66	4.52E-05	1.37E-04
Ndufa12	0.71	1.94E-02	3.61E-02
Ndufa2	0.61	4.61E-14	3.58E-13
Ndufa3	0.73	3.41E-09	1.68E-08
Ndufa4	1.57	1.44E-16	1.32E-15
Ndufa7	0.66	1.52E-18	1.58E-17
Ndufab1	0.75	1.09E-03	2.62E-03
Ndufaf1	0.71	6.61E-04	1.65E-03
Ndufb3	0.56	1.20E-12	8.32E-12
Ndufb5	0.60	3.26E-28	5.34E-27
Ndufc1	0.67	6.38E-05	1.89E-04
Ndufs2	0.72	5.47E-14	4.22E-13
Ndufs3	0.68	3.49E-09	1.72E-08
Ndufs6	0.63	1.70E-09	8.66E-09
Ndufv3	0.53	3.50E-30	6.18E-29
Sdhc	0.60	4.01E-11	2.39E-10
Uqcr	0.60	6.08E-15	5.03E-14
Cox1	0.53	3.42E-04	8.96E-04
Cox7b	0.67	3.17E-12	2.11E-11
Cox7r	0.74	8.98E-05	2.59E-04
Coa5	0.66	4.24E-04	1.09E-03
Cox16	0.66	8.78E-07	3.34E-06
Cox17	0.46	1.09E-14	8.84E-14
Atp5a1	0.75	7.74E-10	4.06E-09
Atp5e	0.65	3.51E-06	1.24E-05
Atp5f1	0.75	9.16E-08	3.88E-07
Atp5g1	0.71	6.68E-06	2.29E-05
Atp5g2	0.71	1.51E-05	4.90E-05
Atp5g3	0.71	5.55E-14	4.28E-13
Atp5h	0.73	2.61E-12	1.75E-11
Atp5i	0.64	1.62E-08	7.46E-08
Atp5j2	0.74	2.73E-07	1.10E-06
Atp5o	0.74	4.03E-10	2.18E-09
Atp5s	0.69	3.09E-03	6.80E-03
Atp6ap2	1.52	2.68E-07	1.08E-06

Beta oxidation	FoldChange	pvalue	padj
Acads	0.52	1.65E-24	2.32E-23
Acadvl	0.61	6.16E-13	4.38E-12
Decr1	0.26	1.23E-94	1.39E-92
Echs1	0.50	8.71E-34	1.80E-32
Eci1	0.49	6.52E-29	1.09E-27
Hadh	0.32	2.79E-66	1.65E-64
Hadha	0.57	9.14E-60	4.63E-58
Hadhb	0.50	6.21E-29	1.04E-27
Mcee	0.73	3.33E-03	7.29E-03
Mut	0.70	2.26E-06	8.19E-06
Pcca	0.48	9.67E-24	1.32E-22

Complement Cascade	FoldChange	pvalue	padj
C1qa	15.75	5.09E-22	6.44E-21
C1qb	21.18	3.73E-22	4.76E-21
C1ra	10.57	5.87E-39	1.49E-37
C2	23.73	7.06E-36	1.58E-34
C3	3.73	1.55E-05	5.00E-05
Cfb	27.29	2.53E-123	4.87E-121
C1qc	14.18	4.65E-30	8.21E-29

Glycolysis	FoldChange	pvalue	padj
Pfkfb4	0.26	7.11E-21	8.50E-20
Ldhb	0.04	3.51E-78	2.67E-76

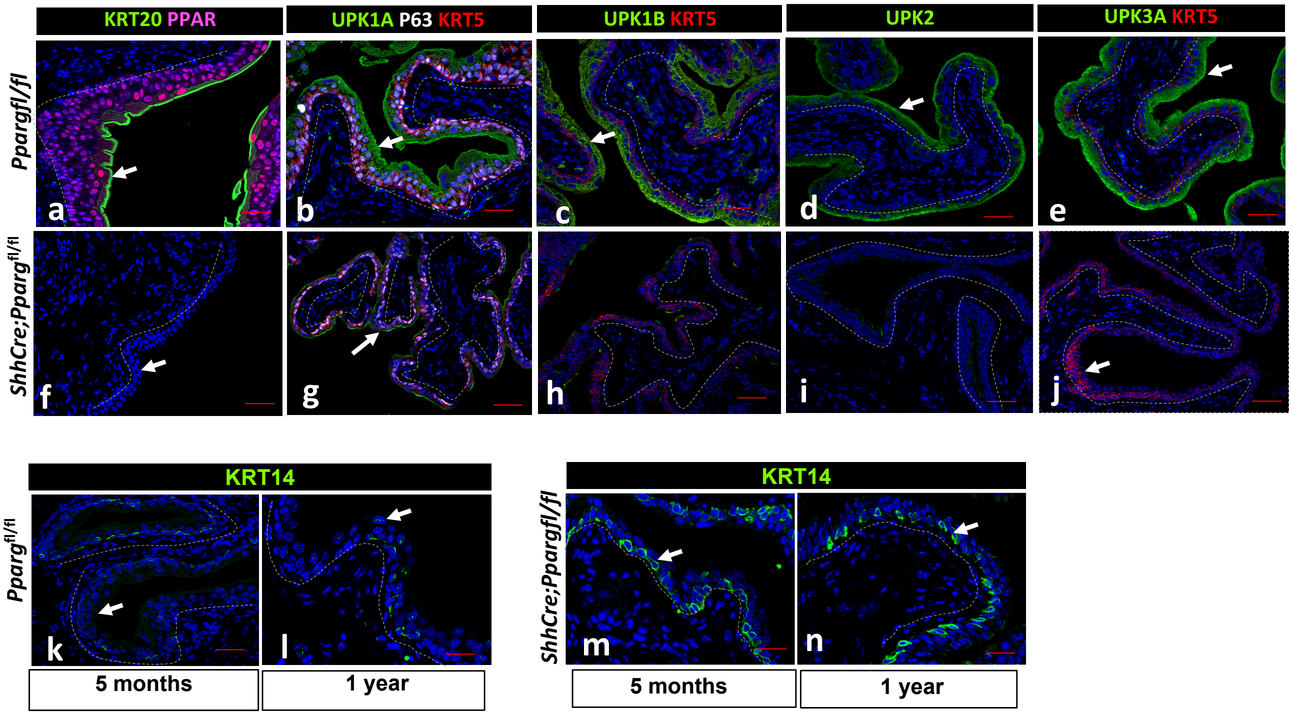

Supplementary Figure 1. Abnormal phenotype in *Pparg*-ablated urothelium. (a, f) Expression of Pparg and Krt20 in adult (a) *Pparg*$^{fl/fl}$ controls and (f) *ShhCre;Pparg*$^{fl/fl}$ mutants. (b, g) Expression of Upk1a, P63 and Krt5 in adult (b) *Pparg*$^{fl/fl}$ controls and (g) *ShhCre;Pparg*$^{fl/fl}$ mutants. (c, h) Expression of Upk1b and Krt5 in adult (c) *Pparg*$^{fl/fl}$ controls and (h) *ShhCre;Pparg*$^{fl/fl}$ mutants. (d, i) Expression of Upk2 in adult (d) *Pparg*$^{fl/fl}$ controls and (i) *ShhCre;Pparg*$^{fl/fl}$ mutants. (e, j) Expression of Upk3a and Krt5 in adult (e) *Pparg*$^{fl/fl}$ controls and (j) *ShhCre;Pparg*$^{fl/fl}$ mutants. (k-n) Expression of Krt14 in 5-month-old and 1-year-old adult (k,l) *Pparg*$^{fl/fl}$ controls and (m,n) *ShhCre;Pparg*$^{fl/fl}$ mutants. Scale bar: 50μm. Adult *Pparg*$^{fl/fl}$ control 5mo; male n=3, female n=3. Adult *ShhCre;Pparg*$^{fl/fl}$ 5mo; male n=4, female n=5, Adult *Pparg*$^{fl/fl}$ control 1y; n=3 Adult *ShhCre;Pparg*$^{fl/fl}$ 1yr; n=3.

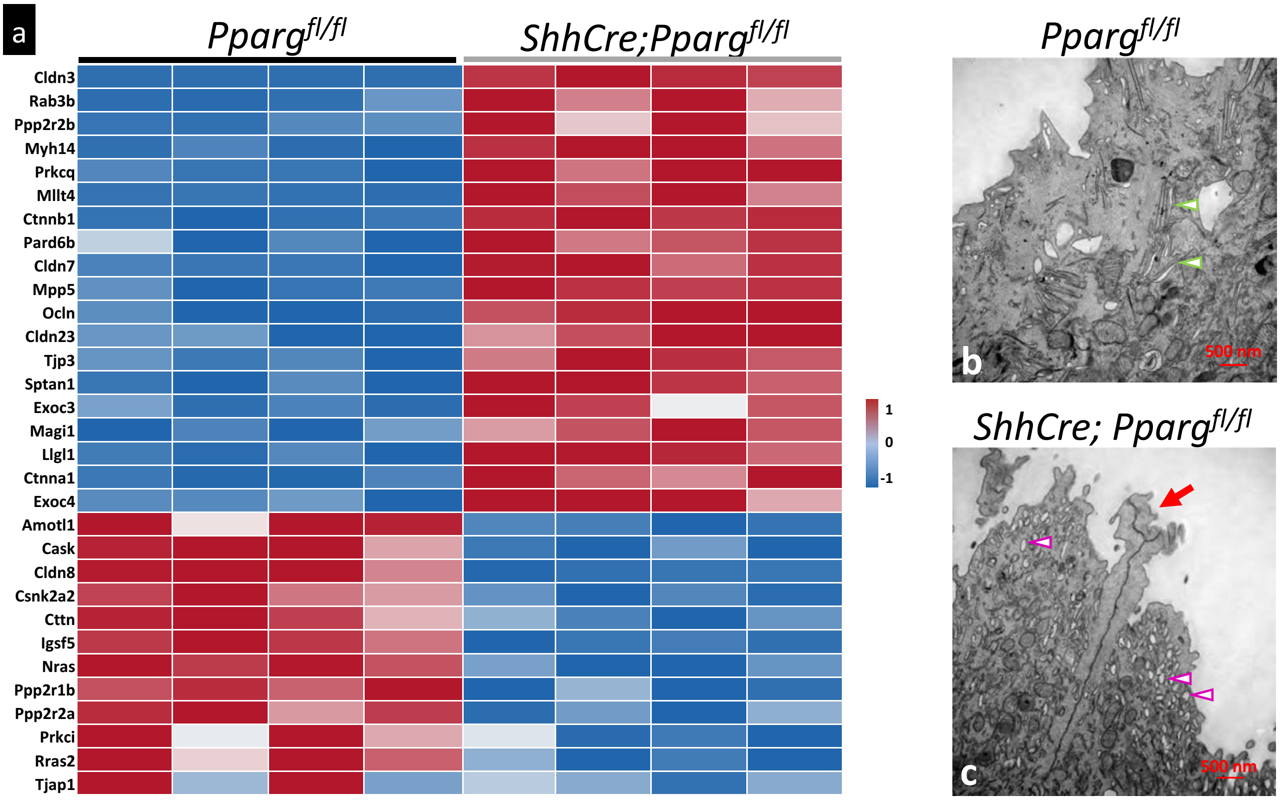

Supplementary Figure 2. Abnormal features in the *Pparg*-mutant urothelium. (a) Heatmap showing the changes in expression of urothelial tight junction genes comparing *ShhCre;Pparg*$^{fl/fl}$ mutants and *Pparg*$^{fl/fl}$ controls. (b-c) Transmission electron microscopy of the urothelium from an adult *Pparg*$^{fl/fl}$ control mouse (b) and a *ShhCre;Pparg*$^{fl/fl}$ mutant (c). Green-white arrowheads in (b) point to specialized transport vesicles in control S-cells. Pink-white arrowheads in (c) point to abnormally shaped vesicles in mutant S-cells. The red arrow in (c) points to an abnormal tight junction between two *ShhCre;Ppargfl/fl* mutant S-cells. Scale bars: 500nm. RNA-seq: *Ppargfl/fl;* n=4, *ShhCrePpargfl/fl;* n=4, Pparg *fl/fl* for EM; n=3, *ShhCrePpargfl/fl* for EM; n=3.

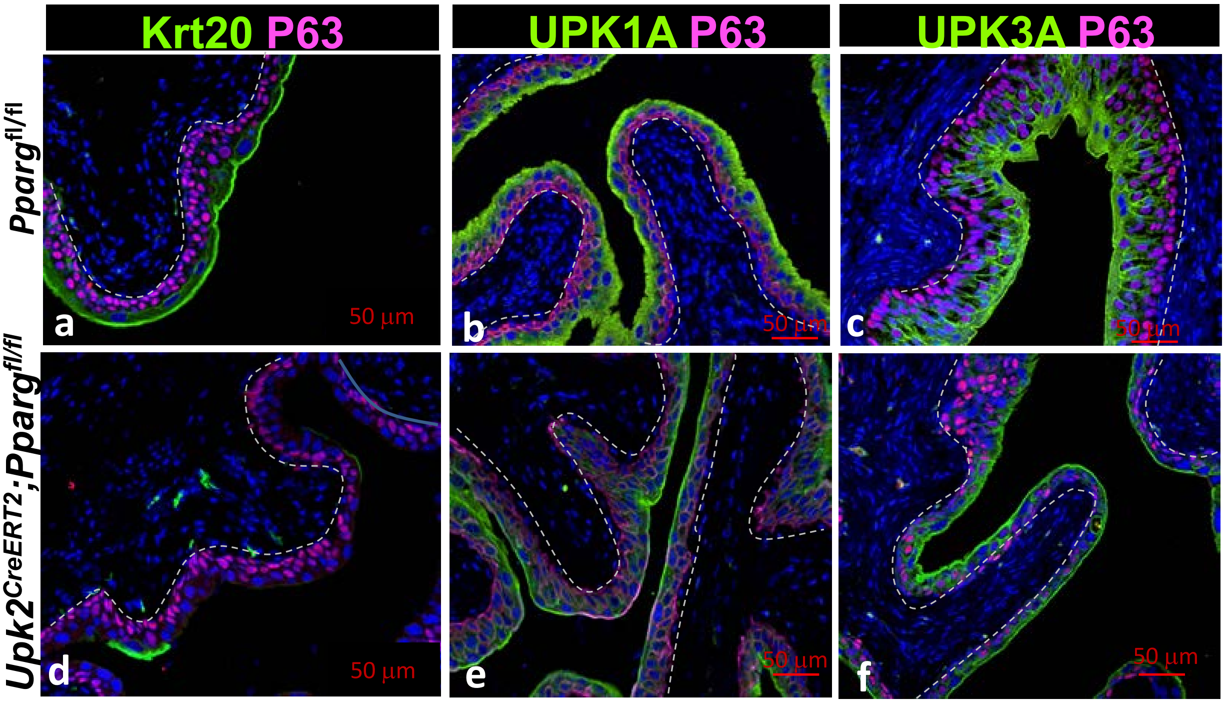

Supplemental Figure 3. Superficial cell markers are down-regulated in *Pparg* mutants. (a-f) Expression of S-cell markers in adult *Pparg^fl/fl^* control mice and *Up2CreERT;Pparg^fl/fl^* mutant mice 7 days after tamoxifen induction. (a,d) Expression of Krt20 and P63 in control (a) compared to a (d) *Up2CreERT;Pparg^fl/fl^* mutant urothelium. (b, e) Upk1a and P63 expression in control (b) and (e) *Up2CreERT;Pparg^fl/fl^* mutant urothelium. (c, f) Upk3a and P63 expression in (c) *Pparg^fl/fl^* control and (f) *Up2CreERT;Pparg^fl/fl^* mutant. Scale bar: 50! m. *Pparg fl/fl* 2wks after tamoxifen; male n=3, female: n=4. *Up2CreERPpargfl/fl* 2wks after tamoxifen; male n=3, female: n=5.

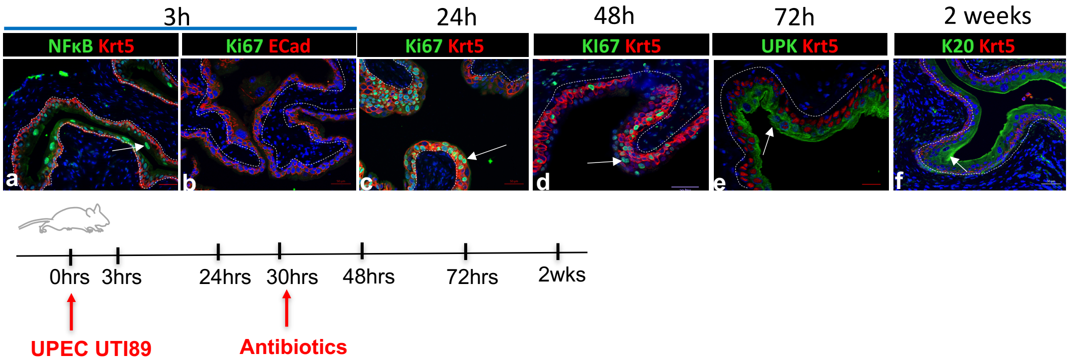

Supplementary Fig. 4: UPEC model of Urinary tract infection. (a) Expression of NF-kB and Krt5 in wild-type adult bladders 3hrs post infection. (b) Expression of Ki67 and E-cadherin 3hrs post infection. (c-d) Expression of Ki67 and Krt5 24hrs and 48hrs post infection. (e) Expression of Upk3a and Krt5 72hrs post infection. (f) Expression of Krt20 and Krt5 2wks post infection. White arrows point to S-cells or cells in the luminal layer. Scale bar: 20μm. n=3 for each UTI time point: n=3. This figure relates to Figure 9 in the main text.

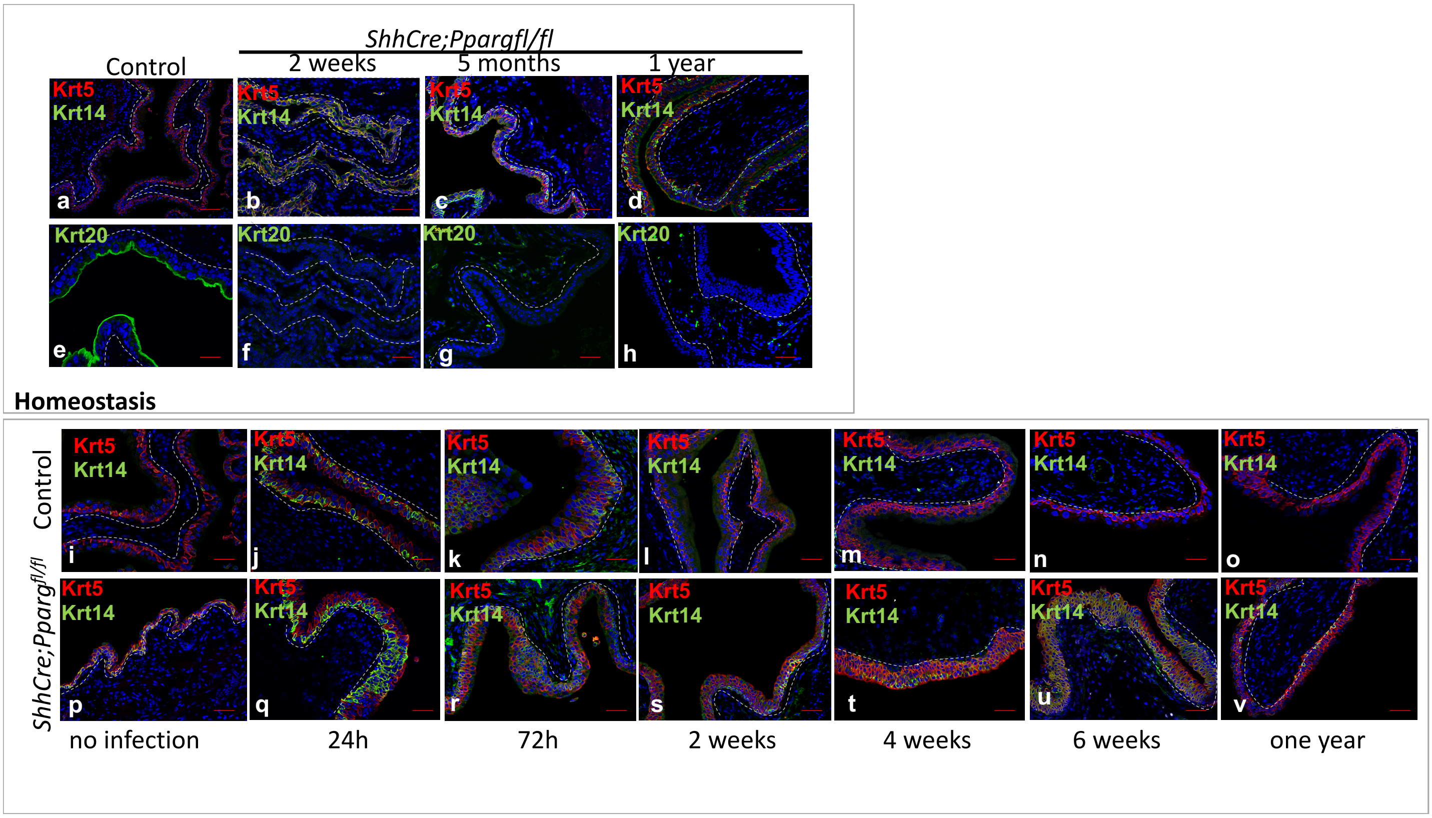

Supplementary Figure 5. Squamous differentiation in *ShhCre;Ppargfl/fl* mutants does not progress to bladder cancer. (a-d) Expression of Krt5 and Krt14 in uninfected (a) *Ppargfl/fl* controls, and in *ShhCre;Ppargfl/f* mutants (b-d) 2 weeks post UTI (b), 5 months post UTI (c), 1 year post-UTI (d). Expression of Krt20 in uninfected (e) *Ppargfl/fl* controls, and in *ShhCre;Ppargfl/fl* mutants (f-h) 2 weeks post UTI (f), 5 months post UIT (g), 1 year post-UTI (h). Expression of Krt5 and Krt14 in control *Ppargfl/fl* mice (i-o) and in *ShhCre;Ppargfl/fl* mutant mice (p-v): (i,p) without infection, (j,q) 24hr after infection, (k,r) 72hr after infection, (l,s) 2wks after infection (m,t) 4wks after infection (n,u) 6wks after infection, (o,v) 1 year post infection. Scale bars=50μm. Adult *Pparg fl/fl* control 2wk; n=3, Adult *ShhCrePpargfl/fl* 2wk; n=3 Adult *Pparg fl/fl* control 5mo; male n=3, female n=3. Adult *ShhCrePpargfl/fl* mutant 5mo; male n=4, female n=5. Adult *Pparg fl/fl* control 1yr; n=3, Adult *ShhCrePparg* 1yr; n=3. UTI 0h Pparg fl/fl control; n=3, *ShhCrePpargfl/fl* mutant; n=3. UTI 24h *Pparg fl/fl*; n=3, *ShhCrePparg fl/fl*; n=3. UTI 72h *Pparg fl/fl* controls; n=4, *ShhCrePparg fl/fl* mutants; n=4. UTI 2wk UTI 4wk *Pparg fl/fl* controls; n=8, ShhCrePparg fl/fl mutants; n=11. UTI 6wk *Pparg fl/fl* controls; n=3, *ShhCrePparg fl/fl* mutants; n=5. UTI 6wk *Pparg fl/fl* control; n=3, *ShhCrePparg fl/fl* mutants; n=5 UTI . 1yr *Pparg fl/fl* controls n=3, *ShhCrePparg fl/fl* mutants; n=4. This figure relates to Figure 9 in the main text.

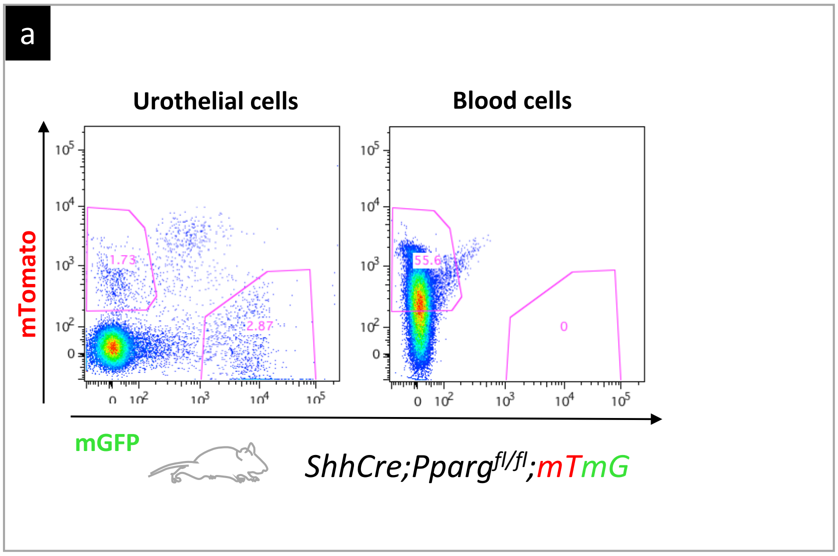

Supplementary Figure 6. *Pparg* is deleted in urothelial cells of *ShhCre;Pparg$^{fl/fl}$* mutants but not in immune cells. (a) To confirm that the *ShhCre* driver is not active in immune cell *ShhCre;Pparg$^{fl/fl}$* mice were crossed with *mTmG$^{fl/fl}$ (Gt(ROSA)26Sortm4(ACTB-tdTomato,-EGFP)Luo/J)* mice (hereafter referred to as *mTmG* mice) to generate *ShhCre;Pparg$^{fl/fl}$;mTmG* mice. In this line, cells undergoing Cre-dependent recombination will express Gfp, and cells that don't undergo recombination will express mTomato. Recombination in the urothelium and in immune cells was assessed by FACS analysis of urothelial cells and immune cells harvested from *ShhCre;Pparg$^{fl/fl}$;mTmG* mutants 24hrs post infection. UTI 24h *Pparg fl/fl* controls; n=3, *ShhCrePparg fl/fl* mutants n=3. This figure relates to Figure 10 in the main text.
